# A Retrospective Test-Negative Case-Control Study to Evaluate Influenza Vaccine Effectiveness in Preventing Influenza Among Immunocompromised Adults With a Solid Organ Transplant

**DOI:** 10.3389/ti.2025.14187

**Published:** 2025-05-16

**Authors:** Manon L. M. Prins, Ernst D. van Dokkum, Aiko P. J. de Vries, Maarten E. Tushuizen, Danny van der Helm, Edwin M. Spithoven, Irene M. van der Meer, J.H. Marc Groeneveld, Leo G. Visser, Saskia le Cessie, Albert M. Vollaard, Geert H. Groeneveld

**Affiliations:** ^1^ LUCID, Subdepartment of Infectious Diseases, Leiden University Medical Center, Leiden, Netherlands; ^2^ Division Acute Internal Medicine, Department of Internal Medicine, Leiden University Medical Center, Leiden, Netherlands; ^3^ Department of Public Health and Primary care, Leiden University Medical Center, Leiden, Netherlands; ^4^ Health Campus The Hague, Leiden University Medical Center, The Hague, Netherlands; ^5^ Leiden Transplant Center, Leiden University Medical Center, Leiden, Netherlands; ^6^ Department of Gastroenterology and Hepatology, Leiden University Medical Center, Leiden, Netherlands; ^7^ Department of Internal Medicine, Amphia Hospital, Breda, Netherlands; ^8^ Department of Nephrology, Haga Teaching Hospital, The Hague, Netherlands; ^9^ Department of Nephrology, Haaglanden Medical Center, The Hague, Netherlands; ^10^ Department of Clinical Epidemiology, Leiden University Medical Center, Leiden, Netherlands; ^11^ Centre for Infectious Disease Control, National Institute for Public Health and the Environment, Bilthoven, Netherlands

**Keywords:** influenza, influenza vaccine effectiveness, influenza vaccination, Netherlands, solid organ transplant patients

## Abstract

Vaccination may prevent influenza in solid organ transplant (SOT) recipients. This study evaluates the influenza vaccine effectiveness (VE) in this high-risk population in the Netherlands. We also compared disease progression and 30-day mortality between vaccinated and unvaccinated influenza patients. In this multicenter, test-negative case-control study, SOT recipients with respiratory symptoms were included when tested for viral respiratory infections during the respiratory seasons between 1 January 2013 and 1 July 2024. Cases had a positive influenza PCR, while controls tested negative. Influenza vaccination in cases (74/174) and controls (291/602) were compared after adjusting for potential confounders. VE was calculated as (1-adjusted odds ratio) x 100. The overall VE was 6.9% (95% CI −40.9 to 38.4), with considerable variation across seasons. For those aged ≥65 years, VE was higher (32.4%, 95% CI −56.5–70.8) compared to those aged 18–64 years (4.8%, 95% CI −56.5 to 42.1). The adjusted VE against influenza A [7.5% (−46.0 to 41.3)] was higher than against influenza B (−3.8% (−146.7 to 56.3)). No differences in influenza-related complications were observed between the vaccinated and unvaccinated cases. The observed seasonal influenza vaccine effectiveness in adult SOT recipients is limited; further investigation for improvement is warranted.

## Graphical Abstract

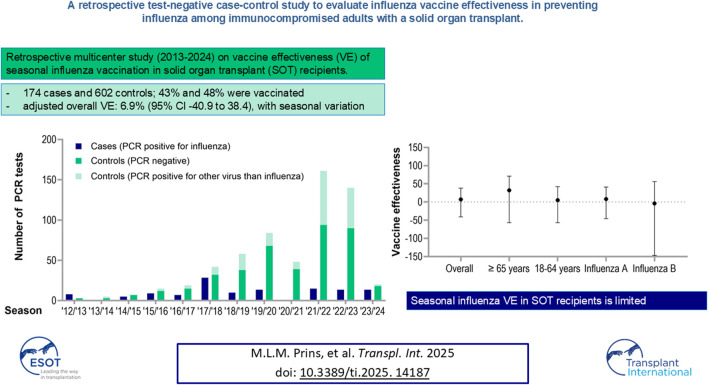

## Introduction

Influenza viruses are globally among the most common causes of respiratory infections in both immunocompetent and immunocompromised individuals, like recipients of a solid organ transplant (SOT) [1]. The prevalence of seasonal influenza among viral pathogens in SOT recipients may vary annually, depending on the types and intensity of circulating viruses, vaccine coverage (i.e., the percentage of a specific population that has received the vaccine), vaccine efficacy related to vaccine-match and dosage of influenza vaccines, type of transplant, and adherence to non-pharmacological interventions [2]. National data from Finland suggests a substantial increased likelihood of detecting laboratory-confirmed influenza and hospitalization due to influenza in kidney transplant recipients compared to the general population [3].

While infection in healthy, immunocompetent individuals may present as a mild and self-limiting condition [4], SOT recipients have an increased risk of influenza-related complications, including secondary bacterial pneumonia, acute graft rejection and mortality [2, 5–8]. Moreover, SOT patients with influenza have a significantly elevated risk of hospitalization, up to 70% [3, 7, 9].

Annual seasonal vaccination is the primary measure for preventing influenza [2] and is universally recommended for SOT recipients [10]. Nevertheless, vaccination rates among SOT recipients are reported to be low in both US and European settings and nearly half of SOT recipients were unvaccinated in registries from the US and Denmark [11, 12].

Lifelong use of immunosuppressive medication affects the lymphocyte function of SOT recipients, thereby leading to an immunocompromised status. Several mechanisms are known, depending on the specific immunosuppressive drug used: reduced T-cell activity, direct suppression of B-cells or antibody production, suppression of cytokine production or inhibition of immune cell proliferation and differentiation. The amount of impairment depends on several factors, such as type of transplant, type of immunosuppression such as mycophenolate or co-stimulation blockers, use of T-cell depleting agents in the year before vaccination and time since transplantation [2, 13]. Consequently, the immunogenicity of the influenza vaccine in SOT recipients is reduced compared to immunocompetent persons, reported as reduced serologic immune responses to influenza vaccines and lower seroprotection rates, based on hemagglutination-inhibition (HI) titers [6, 13–21]. In addition to the immunological (surrogate) marker, two other clinical outcome measures are commonly used for the protective effects of vaccines: vaccine efficacy and vaccine effectiveness (VE). Vaccine efficacy refers to how well a vaccine performs in controlled settings (e.g., clinical trials), while VE describe its performance in real-world conditions. Ultimately the VE is the most relevant outcome. The immune response does not always correlate with the clinical effectiveness of a vaccine. In addition, the VE of the influenza vaccine varies yearly, with mismatches negatively affecting its effectiveness [22]. In the general population, influenza VE ranged from 19% to 59%, with lower percentages among people above 65 years [23–31]. However, studies on the VE of the influenza vaccine in SOT recipients are lacking and therefore its effectiveness remains controversial. In several epidemiological studies, the benefit of influenza vaccination in SOT recipients is only reported in relation to disease progression and the occurrence of complications, such as pneumonia, graft outcomes, intensive care unit (ICU) admission and mortality [9, 12, 19, 32, 33].

The aim of this study is to determine the influenza VE among immunocompromised adult SOT recipients in the Leiden transplantation region in the Netherlands.

## Materials and Methods

### Study Design

We performed a multicenter, retrospective test-negative case-control study [34] to estimate VE of seasonal influenza vaccination in SOT recipients. Patients in the Leiden University Medical Center (LUMC), one of seven transplantation centers in the Netherlands, and its seven affiliated shared-care hospitals (Alrijne Hospital, Amphia Hospital, Groene Hart Hospital, Haga Hospital, Haaglanden Medical Center, Reinier de Graaf Hospital, Spaarne Hospital), were eligible. The study period was between 1st January 2013, and 1st July 2024.

### Study Participants

All adult patients (≥18 years) who received a SOT (kidney, liver, pancreas, islet cells of Langerhans, or a combination of these), and underwent diagnostic testing for influenza in an outpatient setting or within 24 h after hospital admission, were included. Other types of SOT, such as heart or lung transplants, were not included, as these are not performed at the LUMC. The standard protocol in our center mandates SOT recipients to contact the hospital (academic hospital or the nearest affiliated hospital, depending on the duration post-transplantation and the hospital were the patient is monitored) if they experience fever or respiratory symptoms. Influenza diagnostics via polymerase chain reaction (PCR) are readily available during the respiratory virus season in the emergency departments or outpatient clinics. We included only symptomatic patients. The indication for PCR test was determined by the treating physician and hospital.

The respiratory virus season in the Netherlands spans from week 40 in 1 year to week 20 in the following year (early October to mid-May) [35]. Subjects enrolled outside this season were excluded from analysis to avoid bias by calendar time [22]. Patients could be included only once a season, but could be included multiple times if they were tested for influenza during multiple seasons. They were classified as cases if there was at least one positive test during the respiratory virus season; otherwise they were controls. For cases, outcomes up to 30 days following the first positive test were studied, for controls outcomes after the first negative test.

Patients were defined as vaccinated if they had received the seasonal influenza vaccine (standard dose) in the ongoing respiratory virus season, prior to PCR testing. Patients were defined as unvaccinated if no influenza vaccine was received in the current season prior to PCR testing.

### Data Collection

In the Netherlands, the seasonal influenza vaccine, standard-dose trivalent (season 2013/14–2018/19) or quadrivalent (since 2019/20) vaccine, is administered to risk groups by general practitioners (GP), primarily in the months October and November. Influenza vaccination is free of charge. After receiving a standard-dose influenza vaccination, the GP documents the type and date/month of this vaccination in their GP electronic information system. Therefore, data regarding influenza vaccination history was obtained by contacting the patient’s GP, either through a letter/email or by phone. In cases where the vaccination history was not accurately recorded at the GP, the patient was contacted directly. Patients were excluded from analysis if no information was available regarding their vaccination status.

In addition, we retrieved detailed clinical information from the electronic healthcare records, including baseline demographics, test results for (other) respiratory pathogens, comorbidities, and use of immunosuppressive agents. Comorbidity was categorized into cardiovascular disease (CVD), chronic pulmonary disease and diabetes mellitus (DM). The degree of immunosuppression was determined by the type of induction, maintenance and/or rejection therapy. Patients were considered to be highly immunosuppressed if they were treated with triple therapy and/or had received lymphocyte depleting agents (anti-thymocyte globulin and/or alemtuzumab) in the preceding 6 months.

### Outcome Measures

The primary outcome is the adjusted influenza VE over the whole period in preventing the occurrence of laboratory-confirmed influenza in patients with a SOT. Adjusted VE by season, age group and by influenza subtype were also determined. Secondary to this, we compared course of disease (hospital length of stay, ICU-admission, need for mechanical ventilation) and 30-day mortality between vaccinated and unvaccinated lab-confirmed influenza patients.

### Sample Size

The influenza vaccination rate for the entire target population has varied from 50% to 57% in the Netherlands in recent years [36]. The VE in the overall vaccinated population in the Netherlands ranged from 31% to 57% [23, 24]. Based on that data, our hypothesis is that the VE in SOT recipients is around 40%, and the vaccination rate in this group is 50%. This VE corresponds to an odds ratio (OR) of 0.6 and a vaccination rate of 0.375 in the influenza-positive group. Based on an expected case/control ratio of 1/3, the required sample size is 165 cases and 495 controls to detect a VE of 40% with a power of 80% and an alpha of 0.5.

### Statistical Analysis

Continuous variables were reported as means and standard deviations (SD) or as median and interquartile range (IQR), depending on distribution. Categorical variables were reported as numbers and percentages. Baseline differences between groups were evaluated using the independent T-test, Mann-Whitney U test and Chi-squared test, with significance set at p < 0.05. VE was calculated as (1-adjusted OR) x 100% and reported as percentages. The OR is the ratio of the odds of being vaccinated versus not vaccinated with a standard vaccine dosage against influenza among cases and controls. Adjusted ORs and 95% confidence intervals (95% CI) were calculated using multiple logistic regression, with influenza PCR results as the outcome and vaccination status as the primary variable at interest. A univariate logistic regression analysis identified factors independently associated with influenza status, with variables showing p < 0.10 included in the multivariable model (age, history of chronic pulmonary disease, history of rejection therapy, hospital of inclusion, season), alongside clinically relevant factors (use of mycophenolic acid [cell division inhibitors] or highly immunosuppressed status). Incidences were calculated by dividing the number of new influenza cases during a respiratory season by the total number of individuals who underwent organ transplantation at the LUMC and were still alive on January 1 during that season, multiplied by 100. All calculation were made using SPSS statistics 25.0 for Windows.

### Reporting and Ethics

The study was done in accordance with Good Clinical Practice Guidelines. The study was approved by the Institutional Review Board of the LUMC (nWMODIV2_2022034) and the need for informed consent was waived. The study was described according to the STROBE checklist for observational studies.

## Results

**FIGURE 1:**
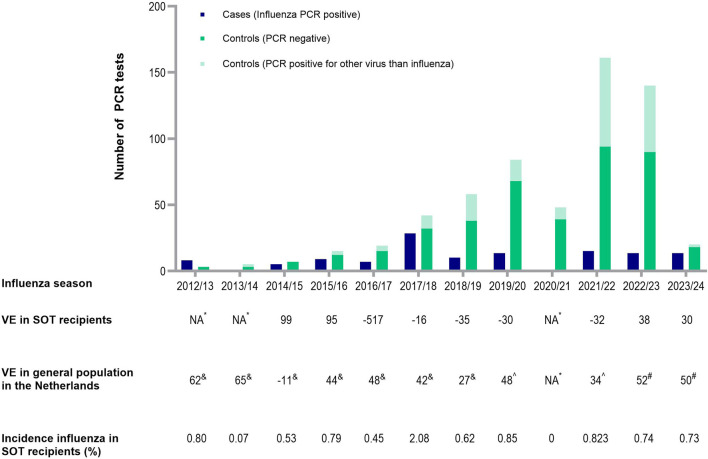
Number of cases and controls, incidence of influenza in SOT recipients and influenza vaccine effectiveness each respiratory season. Presented in the figure are the amount of cases and controls each respiratory season. Below the figure, the adjusted VE in SOT recipients is presented each respiratory year, compared to the yearly influenza VE in the general population in the Netherlands, reported by the National Institute for Public Health and the Environment. In addition, incidence of influenza cases is calculated among all SOT recipients still alive during a respiratory season at January 1 of that season. *NA because no cases were detected (2020/2021) or the sample size was too small (2012/2013, 2013/2014). ^&^Reported by the National Institute for Public Health and the Environment. ^^^Adjusted for the confounders age, history of chronic pulmonary disease, history of rejection therapy, hospital of inclusion, season, use of cell division inhibitors, highly immunosuppressed status. Abbreviations: NA, not applicable; VE, vaccine effectiveness; PCR, polymerase chain reaction; SOT, solid organ transplant.

After excluding 30 patients due to missing vaccination data, 776 participants were included in the analysis: 174 cases and 602 controls. Of all the participants, 207 were included more than once, including 29 cases and 178 controls. Among the controls, 183 had a positive PCR result for another viral pathogen, while 419 patients had a negative result (Figure 1). Of the patients with positive PCR, SARS-CoV-2 (59%), respiratory syncytial virus (16%) and rhinovirus (13%) infections were most common. Most controls underwent PCR testing in 2022 (28.7%), followed by 2023 (15.1%), 2021 (14%) and 2020 (12.6%). Among the cases, 74% tested positive for influenza A and 26% tested positive for influenza B. The influenza A subtype was not determined. Estimated yearly incidence of influenza among transplant recipients is presented in Figure 1 and ranged between 0% (2020/21) and 2.08% (2017/2018).

The demographic characteristics of the participants are presented in Table 1. Cases were slightly younger than controls and the percentage of cases varies by month. Overall, 47% of the participants were vaccinated: 43% of cases (74/174) and 48% of controls (291/602). Among patients aged 65 years and older, 168 out of 365 (46%) were vaccinated, compared to 147/411 (36%) individuals under the age of 65.

**TABLE 1:** Characteristics of patients included in the analysis.

	Overall (n = 776)	Influenza negative/controls (n = 602)	Influenza positive/cases (n = 174)	p^a^
Male sex	459 (59.1)	360 (59.8)	99 (56.9)	0.49
Age, mean (SD)	59.7 (13.4)	60.8 (13.3)	56.2 (13.3)	<0.001
BMI, mean (SD)	25.9 (5.1)	25.8 (5.0)	26.2 (5.6)	0.42
Type of influenza A B	129 (16.6) 45 (5.8)	-	129 (74.1) 45 (25.9)	-
Month of testing January February March April May October November December	149 (19.2) 133 (17.1) 141 (18.2) 89 (11.5) 34 (4.4) 61 (7.9) 66 (8.5) 103 (13.3)	99 (16.4) 92 (15.3) 103 (17.1) 77 (12.8) 32 (5.3) 60 (10.0) 65 (10.8) 74 (12.3)	50 (28.7) 41 (23.6) 38 (21.8) 12 (6.9) 2 (1.1) 1 (0.6) 1 (0.6) 29 (16.7)	<0.001
Pre-existing cardiovascular disease	649 (83.6)	506 (84.1)	143 (82.2)	0.56
Pre-existing lung disease Asthma/COPD Other^b^	227 (29.3) 119 (15.3) 142 (18.3)	186 (30.9) 99 (16.4) 119 (19.8)	41 (23.6) 20 (11.5) 23 (13.2)	0.06 0.11 0.05
Pre-existing diabetes	309 (39.8)	241 (40.0)	68 (39.1)	0.82
Empiric antibiotics	189 (24.4)	155 (25.7)	34 (19.5)	0.09
Time between transplantation and PCR in years, median (IQR)	7 (3–13)	7 (3–13)	6 (2–12)	0.01
Type transplantation Kidney Pancreas Islets of Langerhans Liver Kidney & pancreas Kidney & liver Kidney & islets of Langerhans	642 (82.7) 2 (0.3) 2 (0.3) 105 (13.5) 13 (1.7) 11 (1.4) 1 (0.1)	503 (83.6) 2 (0.3) 1 (0.2) 77 (12.8) 8 (1.3) 10 (1.7) 1 (0.2)	139 (79.9) - 1 (0.6) 28 (16.1) 5 (2.9) 1 (0.6) -	0.41
Type induction^c^ IL-2 inhibitor Alemtuzumab	440 (87.8) 47 (6.1)	336 (88.0) 36 (9.4)	103 (86.6) 12 (10.1)	0.88
No. of Immunosuppressive agents 1 2 3	68 (8.8) 402 (51.8) 305 (39.2)	50 (8.3) 322 (53.5) 229 (38.0)	18 (10.3) 80 (46.0) 76 (43.7)	0.32
Type of immunosuppressive agents Corticosteroids Calcineurin inhibitors Cell division inhibitors MTOR inhibitors Lymphocyte depleting agents	675 (87.0) 612 (78.9) 449 (57.9) 52 (6.7) 48 (6.2)	522 (86.7) 479 (79.6) 343 (57.0) 41 (5.9) 37 (6.1)	153 (87.9) 133 (76.4) 106 (60.9) 11 (6.8) 12 (7.9)	0.67 0.37 0.35 0.82 0.93
Rejection therapy <6 months ago Once Never	151 (19.5) 12 (1.5) 139 (17.9) 625 (80.5)	108 (17.9) 10 (1.7) 98 (16.3) 494 (82.1)	43 (24.7) 2 (1.1) 41 (23.6) 131 (75.3)	0.047
Type of rejection therapy Solumedrol Alemtuzumab ATG Other^d^	124 (16.0) 36 (4.6) 31 (4.0) 39 (5.0)	88 (14.6) 25 (4.2) 21 (3.5) 28 (4.7)	36 (20.7) 11 (6.3) 10 (5.7) 11 (6.3)	0.10 0.26 0.22 0.37
Time between rejection therapy and PCR in years, median (IQR)	6 (2–16)	2 (6–15)	6 (3–18)	0.07
Hospital of inclusion Hospital 1 Hospital 2 Hospital 3 Hospital 4 Hospital 5 Hospital 6 Hospital 7 Hospital 8	26 (3.4) 88 (11.3) 43 (5.5) 171 (22.0) 54 (7.0) 249 (32.1) 45 (5.8) 100 (12.9)	21 (3.5) 78 (13.0) 41 (6.8) 147 (24.4) 47 (7.8) 143 (23.8) 41 (6.8) 84 (14.0)	5 (2.9) 10 (5.7) 2 (1.1) 24 (13.8) 7 (4.0) 106 (60.9) 4 (2.3) 16 (9.2)	<0.001
Vaccinated	365 (47.0)	291 (48.3)	74 (42.5)	0.18
Time between vaccination and PCR in months, mean (SD)	2.8 (1.8)	2.8 (1.8)	2.6 (1.5)	0.53

Data are presented per episode. In total, 207/776 (26.7%) patients were included more than one time. Data are presented as no. (%) unless otherwise indicated.

Abbreviations: IL-2, interleukine-2; SD, standard deviations; IQR, interquartile range; BMI, body mass index; MTOR, mammalian target of rapamycin; ATG, anti-thymocyte globulin.

^a^ Independent T-test, Chi-squared test or Mann-Whitney U test.

^b^ Other types of lung diseases are active lung cancer, bronchiectasis, cystic fibrosis, pulmonal hypertension, sarcoidosis, tuberculosis, obstructive sleep apnea syndrome (OSAS).

^c^ Valid percentages are presented (numbers do not always add up to 776 as there are some missing data).

^d^ Other types of rejection therapy are OKT3 (muromonab), plasmapheresis, IVIG, rituximab, switch to tacrolimus, addition of third agent).

### Overall Vaccine Effectiveness and for Each Individual Season

After adjusting for the previously mentioned confounders, the adjusted VE over the whole period was 6.9% (95% CI -40.9 to 38.4). VE for individual seasons varied widely (Figure 1). Nonetheless, this study was not powered to analyze these yearly VE’s, leading to wide confidence intervals. In the 2020/2021 season, no VE could be determined as no individuals tested positive for influenza. Similarly, VE could not be calculated for the 2012/2013, 2013/2014 and 2014/2015 seasons due to small sample sizes. After excluding this three seasons, the adjusted VE was 4.3% (95% CI −46.6 to 37.5).

### Vaccine Effectiveness by Age Group and by Influenza Virus Type

**FIGURE 2:**
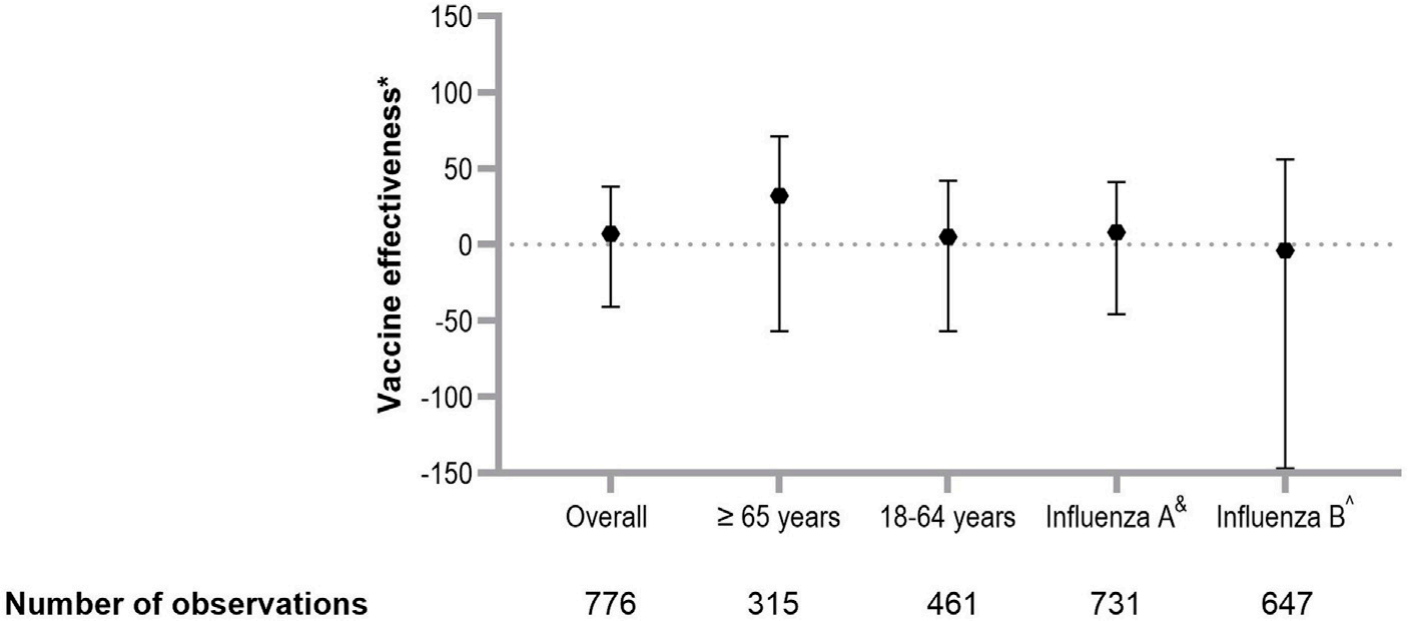
Estimation of vaccine effectiveness against laboratory confirmed influenza. Overall VE in SOT recipients, VE by age group and by influenza virus subtype. Errors bars represent 95% CI. *Corrected for age, history of chronic pulmonary disease, history of rejection therapy, hospital of inclusion, season, use of cell division inhibitors, highly immunosuppressed status. ^&^Only cases with influenza A subtypes were included; cases with influenza B virus subtypes were excluded. ^Only cases with influenza B virus subtypes were included; patients with influenza A virus subtype were excluded. Abbreviations: OR, odds ratio; VE, vaccine effectiveness; CI, confidence interval.

Among individuals aged 18–64 years, the adjusted VE from 2013 to 2024 was 4.8% (95% CI −56.5 to 42.1), compared to a VE of 32.4% (95% CI −56.5–70.8) among those aged 65 years and older (Figure 2). The total adjusted VE against influenza A was 7.5% (95% CI −46.0 to 41.3), while the total adjusted VE against influenza B was −3.8% (95% CI −146.7 to 56.3).

### Course of Disease in Patients Who Tested Positive for Influenza

Overall, 112 influenza-positive patients (64.4%) were hospitalized, with a median stay of 3 days (IQR 2–5 days) (Table 2). Six patients (3.4%) required ICU admission, five of whom needed mechanical ventilation. Overall, the all-cause 30-day mortality among lab-confirmed influenza cases was 1.7%. The course of disease for vaccinated SOT recipients was similar to that of unvaccinated patients. ICU admission, mechanical ventilation, 30-day mortality and treatment for rejection after influenza illness (1.7%) did not differ between vaccinated and unvaccinated patients (Table 2).

**TABLE 2:** Course of disease in patients who tested positive for influenza.

	Overall (n = 174)	Vaccinated (n = 74)	Unvaccinated (n = 100)	p^a^
Admission in the hospital	112 (64.4)	51 (68.9)	61 (61.0)	0.28
Hospital length of stay, median (IQR)	3.0 (2.0–5.0)	3.0 (2.0–4.0)	3.0 (2.0–7.0)	0.25
ICU-admission	6 (3.4)	2 (2.7)	4 (4.0)	0.61
Need for mechanical ventilation	5 (2.9)	2 (2.7)	3 (3.0)	0.92
30-day mortality	3 (1.7)	1 (1.4)	2 (2.0)	0.75
Rejection	2 (1.1)	0	2 (2.0)	0.22

Data are presented as no. (%) unless otherwise indicated.

^a^ Chi-squared test, Fisher’s exact test or Mann-Whitney U test.

## Discussion

In this retrospective test-negative case-control study, the observed adjusted VE against influenza infection of the standard-dose seasonal influenza vaccine in SOT recipients was low over the years 2013–2024 in the Netherlands, with a most optimal adjusted VE of 6.9%. Compared with VE in people below 65 years, the adjusted VE in patients above 65 years was higher (4.8% versus 32.4%, respectively). The VE against influenza B was lower than against influenza A (−3.8% versus 7.5%, respectively). We also showed that influenza-related complications did not differ between the vaccinated and unvaccinated influenza cases.

Data on vaccine effectiveness for preventing influenza infection in adults with immunocompromised status are scarce. Most research has concentrated on assessing the humoral antibody responses by measuring influenza-specific antibody levels, associated with protection in healthy adults, using standard HI assays [37–40]. However, these antibody concentrations are surrogate markers of vaccine efficacy and if these are also protective in SOT recipients is unknown. Therefore, it remains important to determine VE as the primary outcome measure, rather than relying on the immunological response.

Previous immunogenicity studies have reported a lower humoral response to influenza vaccination in SOT recipients compared with healthy controls [15, 18, 21]. Our study is among the first to demonstrate and quantify the clinical impact of this known reduced immunological vaccine response in SOT recipients.

In the Netherlands, the effectiveness of the (inactivated) influenza vaccine ranged from −11% to 65% in the past decade in the general population [23–27]. Our findings suggest that VE against influenza in SOT recipients is low compared to the general healthy population. Similarly, a study by Hughes et al reported an adjusted VE of 5% against influenza-associated hospitalizations among eight categories of immunocompromised adults during the 2017–2018 season, compared to 41% among non-immunocompromised adults [41].

Numerous studies have shown that the estimates of VE in the general population are higher in subjects under the age of 65 years than in those aged 65 years or older [30, 31]. In contrast, we found a higher VE in those aged 65 years or older compared to those aged 18–64 years. This finding aligns with data from the Dutch National Institute for Public Health and the Environment, which also reported higher VE in the older population compared to the younger population [23–27, 42, 43]. A possible explanation could be differences in exposure, healthcare-seeking behavior or disease severity between these age groups. Younger patients with (mild) symptoms may be less likely to seek hospital care than older individuals. This could lead to undocumented mild infections, which might attenuate VE estimates. The low annual incidences of influenza observed in our population, compared to the general Dutch population, supports the idea that there may be more mild cases among vaccinated individuals or high levels of vaccination in household contacts of SOT recipients that may prevent secondary transmission. However, the incidence rates in the general population reflects influenza-like illness (ILI) reported by GP’s, rather than laboratory-confirmed influenza reported by hospitals. Since not everyone with ILI seek hospital care, this may account for the lower incidences of influenza observed in our population.

In earlier influenza seasons, PCR was less widely used than in the (post-) COVID-19 seasons, where PCR on RSV/SARS-CoV-2/influenza was likely done more routinely to all patients with equal severity of disease (who where not tested before COVID pandemic). However, this would not have had an impact on the VE. Lower threshold for PCR testing may result in testing less severely ill patients, resulting in more influenza negative patients (controls). However, the ratio of vaccinated to unvaccinated individuals in a population with fewer cases does not change (as doctors are unaware of the vaccination status of the patient), and the OR and consequently the VE remains unaffected (OR= ((a/b)/(c/d)), where “a” represents the number of vaccinated cases, “b” the number of unvaccinated cases, “c” the number of vaccinated controls, and “d” the number of unvaccinated controls).

Our results showed that influenza-related outcomes -such as hospital length of stay, need for ICU admission and/or mechanical ventilation, 30-day mortality and rejection- did not differ between the vaccinated and unvaccinated influenza cases. However, this only applies to those who presented at the hospital. Due to the retrospective design of the study, we cannot accurately quantify the extend of illness prevented by the influenza vaccine. However, we do instruct SOT recipients to contact the hospital in case of respiratory infection symptoms. Studies evaluating the impact of antecedent influenza vaccination in SOT recipients with influenza disease are scarce. One study that assessed the impact of the 2010–2011 seasonal influenza vaccination on illness severity among SOT recipients with influenza disease reported similar results [19]. The study indicated that receiving the influenza vaccine was not associated with a decreased risk of hospitalization, ICU admission, mortality or severe disease. In contrast to our study, it did find an association with shorter hospital stay. In addition, Kumar et al reported that receiving the influenza vaccine in the current season was associated with a lower incidence of ICU admission in a multivariate model among 616 patients with a SOT or hematopoietic stem cell transplantation [9].

The observed reduced influenza VE in SOT patients in comparison to the healthy population warrants further investigation aimed at improving the VE or investigation to explore alternative strategies to protect this vulnerable group. Various methods had been previously evaluated to improve vaccine immunogenicity in immunocompromised patients, including adjuvanted vaccines [44], the use of high-dose (HD) influenza vaccines [45–48], administration of a booster-dose (BD) [21, 49], intradermal vaccination [50–52] and adjusting immunosuppression to target [53]. Most of these measures have not resulted in clinically significant increases in immunogenicity compared with single standard-dose intramuscular strategies [54]. Of these strategies, HD (especially those four times the standard dose) and BD vaccines seem to be the most promising for enhancing immunogenicity and are generally well tolerated [54].

Several limitations should be considered when interpreting these results. First, the wide confidence intervals surrounding the VE estimate limit the strength of our conclusion. However, the upper bound of the confidence interval still remains below the VE observed in the healthy population. Second, VE fluctuate annually, depending on the degree of antigenic match between vaccine strains and circulating strains [22]. Our study focused on the adjusted VE over 11 respiratory seasons, as yearly sample sizes were insufficient for reliable calculating, introducing some heterogeneity. Third, the observational design of the study also introduces potential confounding. Although we adjusted for all known confounding variables, residual confounding still exist. The test-negative design required that cases seek medical attention, which might not occur for mild symptoms. However, SOT recipients are more likely to contact the hospital for mild symptoms compared to the general population, as they are advised to do so in the presence of fever or symptoms of a viral respiratory infection. Moreover, during the COVID-19 pandemic and the subsequent years, patients were more inclined to seed medical care and get tested for respiratory viruses more readily, which likely mitigates the risk of underestimating VE. Next, the timing of vaccination was not accounted for due to the often unknown exact dates of vaccine administration at many GP offices. Lastly, our criteria for being considered vaccinated were fairly stringent, requiring individuals to have received the seasonal influenza vaccine in current respiratory season before PCR testing. Those vaccinated in the previous season were considered unvaccinated. Less stringent criteria would likely lower the VE estimate, as studies indicate a progressive decline in antibody titers within a year after vaccination [37, 49, 55, 56]. Additionally, VE tends to drop during the season, beginning around 100 days post-vaccination [30]. Thus, vaccinated patients receiving their influenza vaccination longer ago (e.g., those who present to the hospital between May and October) were less protected against influenza disease, which consequently should influence the VE estimate. However, since individuals between week 20 and week 40 were excluded, we believe that the impact of waning immunity on our estimates limited.

The test-negative design represents a strength of our study. By ensuring that all laboratory-confirmed cases and test-negative controls sought care in the same healthcare settings for similar sets of symptoms, we reduce bias related to community-level variations in vaccine coverage. In addition, cases and non-cases will typically originate from the same communities. Another advantage of this design is the reduction in disease misclassification, as cases are confirmed through laboratory testing. Furthermore, we assessed vaccination history by contacting GP’s, who were unaware of their patients’ respiratory infections when verifying vaccination status, thereby reducing misclassification of vaccine history as a potential source of bias. Selection bias, which could arise from physicians’ clinical decision-making regarding testing for influenza, is also mitigated. Since patients’ vaccine history is generally unknown to treating physicians in hospitals- who typically rely on GPs for such records- we further limit potential biases in vaccine status that could affect outcomes.

In conclusion, the results of our study demonstrate that seasonal effectiveness of the standard-dose influenza vaccine against laboratory confirmed influenza in adult SOT recipients is limited. Despite the low precision and limitations of a retrospective analysis, our findings prompt further investigations aimed at improving VE in SOT recipients. New vaccine formulations or a different vaccination strategy may increase VE. In addition, more prospective data with larger sample size on such regional VE estimates are needed, as it could help convince both doctors and patients of the benefits of vaccination. This data collection should not only focus on influenza VE, but also on burden of disease and VE of other vaccine-preventable infections in SOT recipients, such as COVID-19 and RSV. If the low VE and low burden of disease due to influenza were to be confirmed, annual vaccination campaigns focusing on single pathogens may be questioned and use of combination-vaccines including influenza, COVID-19 and RSV would be preferred to limit the number of vaccinations and healthcare consultations.

## Data Availability

The data will be made available on reasonable request.
